# Conceptual Organization is Revealed by Consumer Activity Patterns

**DOI:** 10.1007/s42113-019-00064-9

**Published:** 2019-10-07

**Authors:** Adam N. Hornsby, Thomas Evans, Peter S. Riefer, Rosie Prior, Bradley C. Love

**Affiliations:** 1 University College London, London, UK; 2 dunnhumby, 184 Shepherds Bush Road, London, W6 7NL UK; 3 The Alan Turing Institute, 96 Euston Rd, Kings Cross, London, NW1 2DB UK

**Keywords:** Cognition, Computational social science, Big data, Machine learning, Decision making

## Abstract

Computational models using text corpora have proved useful in understanding the nature of language and human concepts. One appeal of this work is that text, such as from newspaper articles, should reflect human behaviour and conceptual organization outside the laboratory. However, texts do not directly reflect human activity, but instead serve a communicative function and are highly curated or edited to suit an audience. Here, we apply methods devised for text to a data source that directly reflects thousands of individuals’ activity patterns. Using product co-occurrence data from nearly 1.3-m supermarket shopping baskets, we trained a topic model to learn 25 high-level concepts (or *topics*). These topics were found to be comprehensible and coherent by both retail experts and consumers. The topics indicated that human concepts are primarily organized around goals and interactions (e.g. tomatoes go well with vegetables in a salad), rather than their intrinsic features (e.g. defining a tomato by the fact that it has seeds and is fleshy). These results are consistent with the notion that human conceptual knowledge is tailored to support action. Individual differences in the topics sampled predicted basic demographic characteristics. Our findings suggest that human activity patterns can reveal conceptual organization and may give rise to it.

**Electronic supplementary material:**

The online version of this article (10.1007/s42113-019-00064-9) contains supplementary material, which is available to authorized users.

## Introduction

Our everyday experiences shape the way we conceptualize and act in the world. Following this intuition, previous work using text corpora has proved useful in understanding the nature of language and human concepts (Andrews et al. 2009; Deerwester et al. 1990). One appeal of this work is that text, such as from newspaper articles, reflects human behaviour outside the laboratory. However, this text primarily serves a communicative role and is often scraped from curated sources, making it less reflective of real human activity.

In this contribution, we aim to build upon previous work from the text domain by analyzing real-world behaviour from a broad section of the general population as they go about an everyday activity in relative anonymity, namely supermarket shopping. We apply techniques developed in computational linguistics to shopping data from nearly 1.3 million trips. Instead of words and documents, our analyses are over products and shopping baskets. These analyses reveal that human concepts are organized around goals and interactions (e.g. tomatoes go well with vegetables in a salad), rather than their internal features (e.g. defining a tomato by the fact that it has seeds and is fleshy).

Our work speaks to the relative importance of *intrinsic* and *extrinsic* features in concept representation. One way that people may reason about categories is to decompose them into intrinsic features or parts (Plato 1973). On this view, a bird is an animal that typically has wings, feathers, a beak, and so on (Rosch and Mervis 1975). However, extrinsic features are also critical for how humans organize concepts and come to understand the world, to the extent that some concepts may be solely defined by them (Barr and Caplan 1987). For example, Wittgenstein (1967) asserted that the concept of game is undefinable. One might suggest that games are fun, but Russian Roulette is not fun and other activities that are fun are not games. Likewise, not all games are competitive (e.g. Ring Around the Rosie). Instead of defining game in terms of intrinsic features, one solution is to define game relationally—a game is simply something that is played (Markman and Stilwell 2001). Human categories are therefore additionally sensitive to relationships and interactions with other concepts (Markman and Stilwell 2001).

The importance of relations and interactions extends beyond abstract concepts. Many features of concrete concepts are extrinsic (Jones and Love 2007). For example, whilst knowing that tomatoes are taxonomically related to fruits, people commonly associate them with other vegetables. Even for natural kinds, people commonly list extrinsic features for concepts (Jones and Love 2007), such as noting that birds eat worms. Meanings appear to update in light of extrinsic relationships. For example, people are more likely to judge a polar bear and a dog as similar after reading vignettes in which both played the same role in a relation, such as chasing some other animal (Jones and Love 2007). Likewise, merely sharing a thematic relationship, such as a man and a tie (e.g. wears), makes the linked concepts more similar (Schank and Abelson 1977; Wisniewski and Bassok 1999; Jones and Love 2007).

When concepts are defined in terms of other concepts, what moors or grounds our concepts to the physical world we inhabit (Harnad 1990)? One proposed solution is that some concepts are embodied (Barsalou 2008). For example, the action of hammering may be grounded to related motor programmes and associated perceptions, linking the body, mind, and physical world. Indeed, neuroscientific evidence has shown that comprehension of language is tightly coupled with the neural regions associated with action and perception (Pickering and Garrod 2013). A computational model developed by Mitchell et al. (2008) was able to accurately predict the neural activity elicited by a noun by considering the co-occurrence of that noun with action verbs in a large-text corpus. In effect, the action verbs, for which elicited neural activity was known, provided a grounding or bases for representing associated nouns.

These corpus models, such as *Latent Semantic Analysis*, use the co-occurrence of words within some context (e.g. a document) to learn lower dimensional, vector representations of word concepts (Deerwester et al. 1990). Like the reviewed psychological research (Jones and Love 2007), words need not directly co-occur with one another to become more similar, but need only occur in similar contexts. Although LSA has enjoyed numerous successes, cases in which its representations diverge with those of humans has prompted further model development (Wandmacher et al. 2008).

One subsequent proposal, *Latent Dirichlet Allocation (LDA)*, is a probabilistic approach in which documents are generated according to a mixture of probabilities over latent themes or topics (Blei et al. 2003). For example, LDA may find that the words ‘Parliament’ and ‘Prime Minister’ have a high probability of belonging to the same topic (e.g. ‘politics’). A passage about the Prime Minister visiting the Houses of Parliament would make this politics topic highly probable, though other topics would also be somewhat likely, such as a topic related to tourism (Big Ben is part of the Houses of Parliament).

The representations learned by topic models appear similar to the concepts that people use (Griffiths et al. 2007; Andrews et al. 2009). For example, topic modelling can predict subsequent words in a sentence, disambiguate word meanings, and extract the gist of a sentence (Griffiths et al. 2007). Related techniques find that word meanings extracted for text corpora reflect back that society’s gender stereotypes (Bolukbasi et al. 2016). These successes emphasize the importance of extrinsic roles and relationships.

People learn thematic relations by observing co-occurence in events or situations (Estes et al. 2011). In corpus analysis, word co-occurrence in language is assumed to be a proxy for co-occurrence in the wild. However, this assumption may not always hold. For example, words can co-occur in language without being semantically related (e.g. *iceburg*→ *lettuce*). More generally, most spoken language is concerned with effective communication of relevant information (Grice 1975), rather than providing a faithful record of object interactions. For example, in waiting to cross the street with a companion, one would never verbalize that the passing car drives on the road. Written language also tends to be curated. For example, journalists adhere to particular guidelines and aim to report on stories of interest to their readership. Whether it’s from natural language or otherwise, data that captures co-occurrence of events in the wild is best suited to evaluate the structure of people’s thematic representations.

**Fig. 1:**
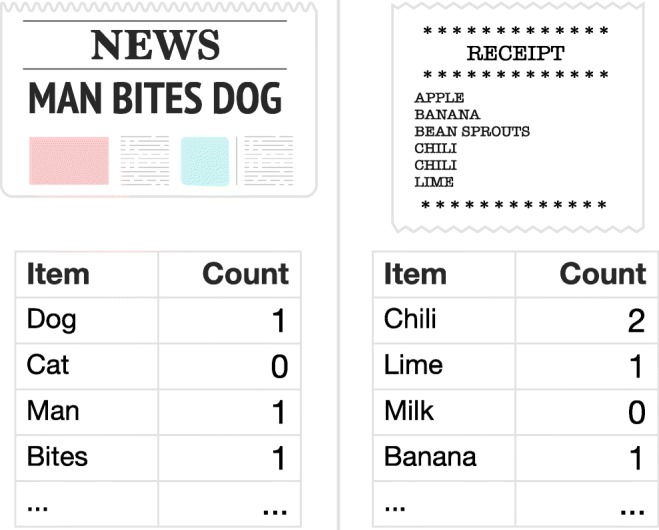
The input in a corpus analysis is typically item counts (i.e. word counts) within some context (e.g. a sentence or document). Analogously, products (akin to words) are organized into baskets (akin to sentences). One advantage of applying these analysis techniques to baskets is that, unlike natural language, meaning is unaffected by item order

An alternative dataset that may help to further evaluate the influence of extrinsic features on people’s representations is consumer retail data. Retail data are collected from consumers as they purchase products together in the same basket, analogous to how words group together in the same document (see Fig. 1). Whilst a person may be conscious not to voice every item they bought in their supermarket shop, one’s grocery receipt provides a faithful record of what they purchased in a supermarket visit. Importantly, this data is traceable to an individual, which contrasts with most corpora analyses, which tends to be based on language in newspapers and books (e.g. Griffiths et al. 2007). Large-scale analyses of grocery retail data is therefore well placed to evaluate the claim that individual differences in people’s experience of the world leads them to possess different thematic representations. In particular, it may help to supplement existing research investigating how people cross-classify food (Murphy and Ross 1999; Ross and Murphy 1999; Lawson et al. 2017; Blake 2008), such as elucidating how regional and generational differences affect people’s thematic representations.

An additional benefit of using consumer purchasing data is that it suits the mathematical assumptions of topic models particularly well. For example, natural language researchers typically use their domain expertise to remove function or ‘stop’ words that have little semantic meaning (such as *the, of, and*). They may also ‘stem’ words to remove prefixes and suffixes of words that have similar semantic meaning (e.g. eat vs. eating). Moreover, the order of words in sentences can also make a big difference to sentence meaning (e.g. ‘dog bites man’ vs. ‘man bites dog’). However, most standard implementations of topic models (based on the original algorithm by Blei et al. 2003) typically ignore word order, instead preferring to consider language as a ‘bag-of-words’ (for an alternative, see Huang and Wu 2015). In contrast, for retail data captured in-store, there is no inherent order for products within a basket, nor a need to remove stop words or perform stemming.

**Fig. 2:**
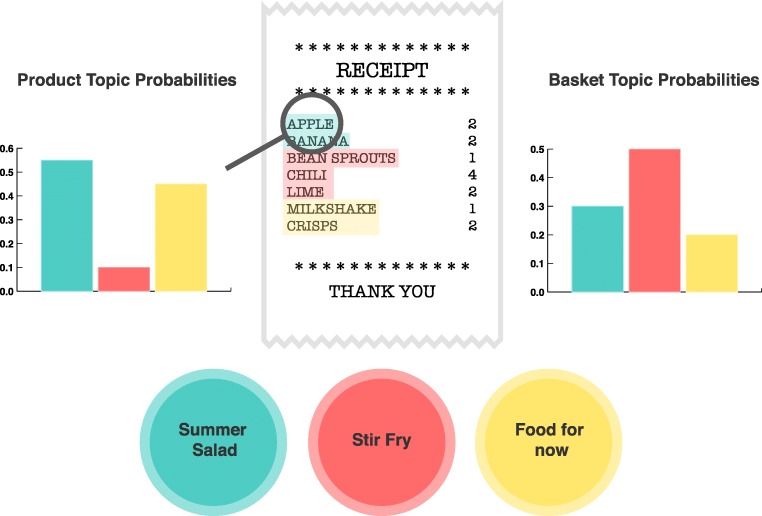
Latent Dirichlet Allocation (LDA) uncovers the higher-level product topics that can be viewed as generating the observed baskets purchased by consumers. LDA’s fit is driven by the co-occurrence pattern of products within baskets. In the solution, each product has a probability of occurring within each topic (shown on the left for apple). The colours illustrate which topic each product would have been labelled with if using the maximum product topic probability. Each basket is generated by a mixture of probabilities over the topics (shown on the right for this basket)

If people’s thematic organization of concepts arises through their interaction with the environment, then it should be possible for a topic model to recover relevant representations of these through consumer purchasing patterns, as shown in Fig. 2. Whilst earlier research has indicated that this is possible, none (to this author’s knowledge) have explicitly measured the likeness of learned topics to consumer’s mental representations (Iwata and Sawada 2013; Iwata et al. 2009; Hruschka 2014). Although people have been shown to default to a taxonomic organization (e.g. tomato → fruit) when asked to freely sort food items in the lab, the presence of a goal can lead to a thematic organization (e.g. tomato → salad) during decision-making (Murphy and Ross 1999; Ross and Murphy 1999). Because shopping is highly goal-directed, we hypothesized that the topics recovered by a topic model would reflect thematic organization. We tested these predictions using a large, anonymized dataset of 1,252,963 shopping baskets and 5,753 unique products, suppliedby one of the UK’s largest supermarket retailers. After optimizing an LDA solution using fit statistics and checking for convergence,^1^ we labelled the 25 topics recovered by the model.

To foreshadow, we found that LDA recovered meaningful topics that were primarily goal-directed and thematic in nature. We confirmed the psychological reality of these topics in two human studies, one with judgments from retail experts and another involving typical consumers. Further support came from analyses showing that topics tied to a season varied sensibly in their prevalence over the calendar year (e.g. the Christmas topic was most prevalent in December). Overall, these results suggest that—contrary to early research on cross-classification of food (Murphy and Ross 1999; Ross and Murphy 1999)—thematic relations dominate representations of food. This is in line with more recent claims that thematic relations may be more numerous than taxonomic associations in people’s stored semantic network and may be more easily revealed when examined at scale (Estes et al. 2011; Lawson et al. 2017). Final analyses tested whether an individual’s shopping experience shaped their conceptual organization of the products. In support of this assertion, the rate at which an individual sampled topics (based on recent shopping history) predicted the individual’s age, gender, and geographic region. This suggests that individual differences in people’s experience can lead them to possess notably different thematic representations from each other. This is important, because it suggests that food-related themes discussed in the literature are likely a function of the participants’ individual experiences and culture.

## Training a Topic Model with Retail Data

### Method

#### Data

The topic model was developed on a random 0.1% sample of all grocery transactions that occurred in 2014 in one the UK’s largest supermarket chains. The transactions were filtered such that only relatively popular products selling > 50,000 units annually were kept. Moreover, data was filtered such that only large baskets containing ≥ 20 items were kept. Filtering was performed to ensure that LDA would have enough observations to learn meaningful topics. This is typical in LDA modelling (Yan et al. 2013) and is performed by the original LDA authors (Blei et al. 2003). After filtering, the final dataset contained 1,253,183 unique baskets and 5753 unique products.

Items were modelled at the product code level. Concretely, there is a different product code for each distinct product in the supermarket. Small variations in that product (i.e. different sizes of the same t shirt) are not given separate codes however.

Note that—unlike traditional uses of LDA in NLP—we did not remove commonly occurring items from documents (i.e. ‘stop words’). Whilst natural language may contain ‘stop words’ (i.e. common words with little semantic meaning such as ‘the’), we did not believe grocery transactions to suffer from the same problem. In the retail case, purchasing popular products, such as milk, bananas and bread, may be informative, perhaps indicating that the consumer is stocking essential items. The basket data was fully anonymised for general research purposes so as to not be personally identifiable.

### Model Fit

In our experiments we applied Latent Dirichlet Allocation (LDA) to the data, using the machine learning library in Spark 1.6.0 (Apache Software Foundation 2016). We conducted a range of experiments to identify the optimal set of hyperparameters (including the number of topics *k*) and in each case monitored the training and test log-perplexity to ensure model convergence and generalization, respectively (see Supplemental for further details).

The LDA solution with the lowest log-perplexity on held out data (i.e. best generalization) had 25 topics. Models were trained for a maximum of 500 epochs, used the Online Variational Bayes optimization algorithm with an *α* = 0.1. The remaining hyperparameters were set to the package defaults.

### Results and Discussion

The topics recovered by LDA were coherent and readily labelled by the authors. Table 1 shows the top 5 products within each of 10 randomly selected topics, according to the relevancy metric. Topics tended to be organized along activity patterns and goals, ranging from specific (e.g. *Stir Fry*) to general in scope (e.g. *Cooking from scratch*). This therefore provides early support for the hypothesis that consumers primarily recruit thematic representations when conducting their grocery shop.

**Table 1:** Retailer-supplied product descriptions for the 5 most relevant products within each of the 10 surveyed topics. Note that the authors had access to the full product topic relevancy matrix (see https://osf.io/tsymx/) when they labeled the topics. Brand names have been removed from this table for publication

Topic	Description
Food for now	ITALIAN BEEF LASAGNE 450G
	ITAL CHICKEN & BACON PASTA BAKE 450G
	ITALIAN MACARONI CHEESE PASTA 450G
	ITAL SPAGHETTI CARBONARA 450G
	ITAL HAM & MUSHROOM TAGLIATELLE 450G
Summer salad	BUNCHED SPRING ONIONS 100G
	ICEBERG LETTUCE EACH
	WHOLE CUCUMBER EACH
	SALAD TOMATOES 6 PACK
	GROWING SALAD CRESS EACH
Stir fry	FRESH EGG NOODLES 375G
	VEGETABLE & BEANSPROUSTIR FRY 333G
	CHINESE STIR FRY BOWL 300G
	EXPRESS GOLDEN VEG RICE 250G
	BEANSPROUTS 370G
Afternoon tea	2 EGG CUSTARD TARTS 2X90G
	BRS/SKIMMED MLK 1.136L/2PINTS
	DANISH SLICED WHITE BREAD 400G
	MINHUMBUGS 200G
	BANANAS LOOSE
Loose fruit and veg	CARROTS LOOSE
	BANANAS LOOSE
	PARSNIPS LOOSE
	CONFERENCE PEARS LOOSE
	BROCCOLI LOOSE
Low calorie options	LIGHFRUITS YOGUR6X175G
	BRSKIMMED MILK 2.272L/4 PINTS
	LIGHYELLOW FRUIYOGUR6X175
	LIGHTOFFEE YOGUR175G
	LIGHLIMITED EDITION YOGHURT 165G
Cheapest option	EDAY VALUEBAKED BEANS IN TOMSAUCE 420G
	EVERYDAY VALUE HAM 364G
	EDAY VALUE MILK CHOCOLATE DIGESTIVES 300G
	EDAY VALUEPENNE 500G
	EVERYDAY VALUELOW FAFRUIYOG 4X125G
Cooking from scratch	COURGETTES LOOSE
	LOOSE BROWN ONIONS
	RED ONIONS LOOSE
	CARROTS LOOSE
	GARLIC EACH
Christmas	ORIGINAL CRISPS 190G
	SOUR CREAM & ONION CRISPS 190G
	BRUSSELS SPROUTS 500G
	PARSNIPS PACK 500G
	SAL& VINEGAR CRISPS 190G
Low maintenance cooking	PREPARED BABY SPROUTS 180G
	PREPARED CARROCAULIFLOWER & BROCCOLI 370G
	PREPARED TRAD SLICED RUNNER BEANS 185G
	PREPARED BROCCOLI FLORETS 240G
	TOPSIDE OF ROASTBEEF 85G

When proposing labels for the topics, the authors had access to the full item-topic relevancy matrix. In some cases, products outside of the top five were instrumental in determining the topic label. For example, mince pies (a popular dessert consumed during Christmas in the UK) were the seventh most relevant item within the *Christmas* topic and chicken korma was eighth most relevant within the *food for now* topic.

## Evaluating Topic Labels with Retail Experts

To evaluate the appropriateness of the topic labels, we conducted a more detailed study. Specifically, a group of industry experts were asked to look through a sample of highly ranking products from within 10 randomly selected topics and confirm that the proposed labels were indeed representative of the grouped products.^2^

### Method

#### Participants

Participants were recruited internally within the UK headquarters of dunnhumby (www.dunnhumby.com), a customer marketing company with over 29 years of experience working with grocery retailers and fast moving consumer goods (FMCG) brands. Employees were asked to participate via the company intranet and were not remunerated. Fifty-one participated in the study. Participants had a wide range of roles within the business, including data analysts, category experts, company directors and client leads. Of these, 56.86% were male. Participants were surveyed in early December 2016 and were blind to the purposes of this study. The Ethics Committee at the UCL Experimental Psychology department approved the methodology and all participants consented to participation through an online consent form at the beginning of the survey.

#### Materials

The study was hosted on an internal company server. Participants accessed the study via their web browsers and answered questions by clicking on the appropriate radio button with their cursor. The study was 1700 × 1300 pixels within the browser.

In each trial, participants were shown 10 product images (2 rows of 5) and accompanying product descriptions from a single topic. Images were 540 × 540 pixels each. Descriptions appeared below each image in size 12 font. The displayed products were the 10 with the highest *relevance*.^3^ Product descriptions and images were downloaded from the retailer’s website in late November 2016.

#### Design

All participants were asked to label the same 10 topics in a random order. The dependent measure was the proportion of times that participants selected the topic label originally proposed during the model development phase. This proportion was then compared against a random baseline, to check whether participants were responding non-randomly.

It was not feasible to survey participants about all 25 topics in the final LDA solution given constraints on employee time. Therefore, 10 topics were chosen from the original 25 to include in the survey.

#### Procedure

Participants were first briefed about the purpose of the experiment. After agreeing, they were then asked to label a group of products for 10 separate topics. Four possible labels were suggested using radio buttons. The order of the presented topics was randomized. One of the four labels was the ‘target’ label proposed by the authors whereas the other three were randomly selected from the remaining nine topics. After selecting a topic label, participants then confirmed their choice with a ‘Continue’ button, before seeing the next set of products from a randomly selected remaining topic. At the end of the study, participants were debriefed.

### Results and Discussion

**Fig. 3:**
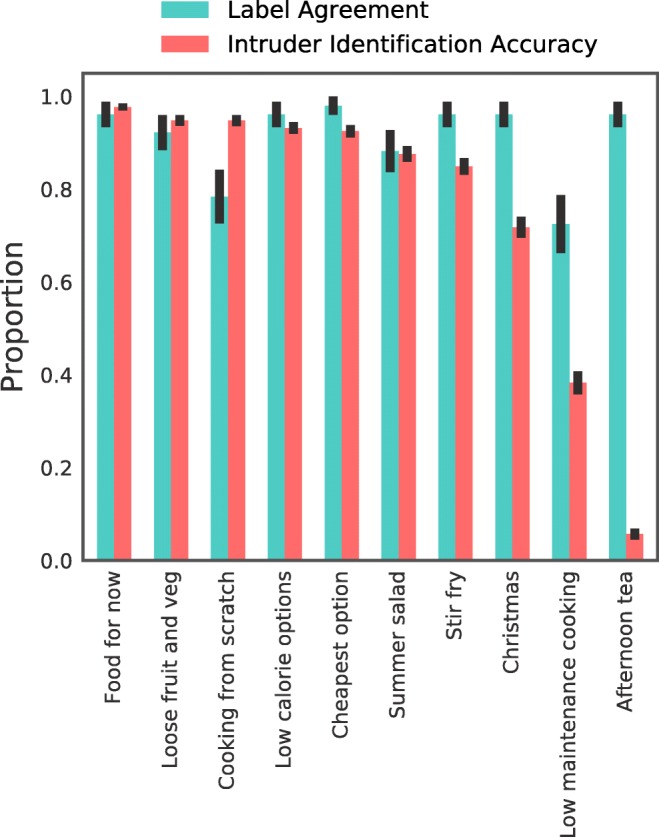
Proportion correct with standard error bars for the study on label agreement involving retail experts and the intruder study involving typical consumers. All proportions were significantly different (*p* < .001) than chance levels, 25.00% (1 of 4) and 16.67% (1 of 6), respectively

When asked to select the appropriate topic label for a group of products from a list of four possible labels, 92.8% (*SE*= 0.015) of the 51 retail industry experts selected the same topic label as was originally proposed by the authors. A two-sided binomial test showed this to be significantly above chance (*p* < .001). Figure 3 shows the proportion of times participants agreed with the originally proposed topic label for each topic.

This high-level of accuracy from a group of experts, who were naive to our research programme, indicates that the topics recovered from consumer activity patterns are meaningful. Disagreement regarding topic labels was primarily driven by conceptually similar topics (further details of this are available in the Supplemental). For example, the most common error in labeling the *summer salad* topic was *cooking from scratch*. These errors are reasonable and are also consistent with the notion that baskets are generated by a mix of topics, as opposed to a single topic (see Fig. 2).

## Seasonal Trends in Topics

The results of the expert study discussed in the “Evaluating Topic Labels with Retail Experts” section suggested that the names given to the topics were reasonable. As further confirmation of this, we attempted to evaluate the appropriateness of names pertaining to seasonal events using historic data. Specifically, we identified 4 topics that were likely to have a highly seasonal popularity (*summer fruits*, *summer salad*, *Christmas* and *low calorie options*) and 4 ‘staple-food’ topics that we believed unlikely to vary as much over the year (*loose fruit and veg*, *Northern Ireland*, *quick to prepare meals* and *food for now*).

### Method

#### Data

To evaluate seasonal trends in topic prevalence, the same data used in the “Training a Topic Model with Retail Data” section was used.

#### Analyses

To calculate the monthly prevalence of each topic, we hard-assigned each basket to belong to one topic, using the maximum topic probability. We then calculated an index indicating the relative popularity of a topic in a given month by calculating the proportion of baskets belonging to a given topic in a month and dividing it by the average topic probability for a given month across all topics.

### Results

**Fig. 4:**
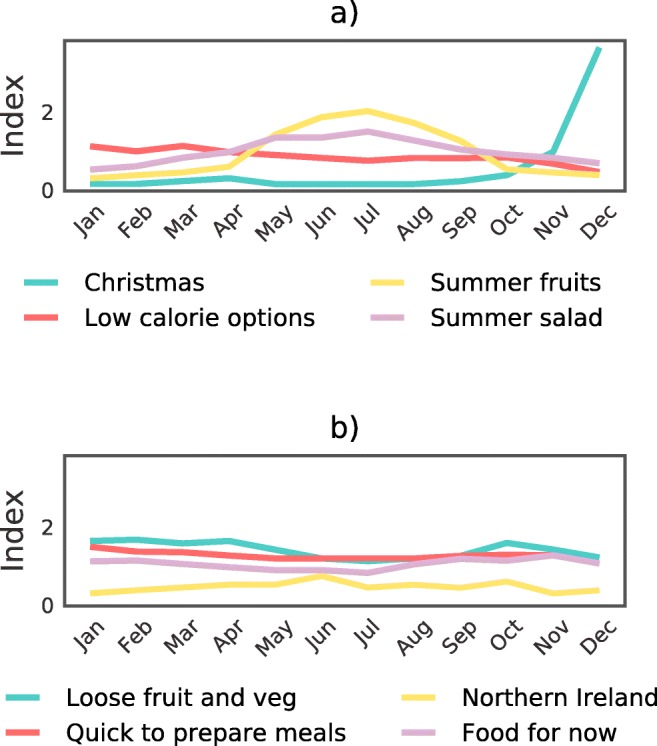
Topic prevalence varies by season. The proportion of baskets with a given topic label in each month of 2014, divided by the monthly mean average across all topics (i.e. index), is shown. **a** Topics that should be seasonal peak at the expected time, such December for the Christmas topic. **b** In contrast, topics for staple products vary less in prevalence over time

Figure 4 shows the popularity of several topics in each month of 2014 in one of the UK’s largest retailers. In line with our hypotheses, *summer fruits* and *summer salad* peaked in popularity during the summer months. Contrasting, baskets labelled with the *Christmas* topic peaked in popularity during December and the surrounding winter months. *Low calorie options* appeared to peak in January and steadily decline to its lowest level of popularity in December. The ‘staple’ topics shown in Fig. 4b appeared to vary considerably less over the year compared to the more seasonal topics. These results give further credence to the proposed topic labels and illuminate some seasonal variations in behavioural patterns that likely reflect time-dependent characteristics of people’s thematic representations. Similarly intuitive patterns were shown to occur during different days of the week, which are reported in more detail in the Supplemental.

Results from the survey of retail experts (in the “Evaluating Topic Labels with Retail Experts” section) and these seasonal analyses support the validity of the topic labels assigned by the experimenters. This therefore provides further credence to the hypothesis that people’s representations are dominated by thematic categories during shopping. It is particularly exciting that this can be inferred using data collected from consumers activity patterns in the wild. By combining big behavioural datasets with computational modelling in this way, we are able to inferences about people’s semantic representations on a far greater scale than would be possible using standard experimental tasks (e.g. card-sorting or free association tasks) (e.g. Murphy and Ross 1999). However, these analyses alone do not confirm the psychological reality of the representations found by this topic model alone. Specifically, there is still an open possibility that the topics recovered by LDA are only meaningful to commercial retail experts, and thus not real consumers.

## Evaluating Topic Coherency with Typical Consumers

To understand whether the product representations identified by the topic model were meaningful to real consumers, we conducted a controlled experiment. Specifically, a large sample of British supermarket shoppers were shown a set of products; 5 of which were highly ranked from within a topic and one that was an ‘intruder’ product, randomly selected from one of the other topics. Participants were asked to select the intruding product. This paradigm is often used when evaluating the fit of computational models to human semantic memory (De Deyne et al. 2016). It was hypothesized that if topics were representative of the mental categories held by consumers, participants would be able to identify the intruding products significantly above chance levels.

### Method

#### Participants

Participants were recruited using dunnhumby’s consumer survey panel; Shopper Thoughts (https://shopperthoughts.com/). Participants completed the survey as part of a larger, monthly survey for 50 card loyalty points. The final sample consisted of 3840 participants, of which 59.47% were female. The modal age group was 55-59 (*n* = 501) and 724 participants did not disclose their age. All participants were from England, Scotland or Wales with the majority of respondents based in central England (*n* = 946). Participants were surveyed during March 2017. The Ethics Committee at the UCL Experimental Psychology department approved the methodology and all participants consented to participation through an online consent form at the beginning of the survey.

#### Materials

The study was accessible via the web, after logging in to the survey platform. The study screen was 960 × 455 pixels. Product images and descriptions were the same as as those described in the “Evaluating Topic Labels with Retail Experts” section. Participants responded to the survey by clicking on a radio button next to the picture and product description of the item they believed to be the intruder.

#### Procedure

Participants were first informed that the purpose of the study was to help retailers group together products found in the supermarket. They were then informed about the study’s procedure. After agreeing to participate, the sole trial started.

Participants were shown six images of products (two rows of three) alongside product descriptions. Five were the most relevant from within a topic and the other ‘intruder’ was the most relevant product from a randomly selected alternative topic.^4^ Participants were asked to ‘spot the one that does not belong in the group’ by clicking on the appropriate radio button underneath the image. Following their choice, participants were thanked, debriefed about the purpose of the research and remunerated immediately.

#### Design

The dependent variable was the proportion of times that participants identified the intruder product. This proportion was then compared against a random baseline to assess whether participants were able to identify intruders significantly above chance levels.

Participants each completed one trial in which they were asked to identify the intruding product. Participants were randomly selected to see one of the 10 topics also used in the retail experts study. This ensured that comparisons between the two related studies were consistent.

### Results and Discussion

Of the 3840 British consumers surveyed, 74.1% (*SE* = 0.007) were able to correctly identify the intruder product. A two-sided binomial test showed this to be significantly above chance (*p* < .001). Figure 3 shows accuracy by topic.

One topic stands out for its below chance level of performance, *afternoon tea*. Participants were most likely (51.7% of the time) to incorrectly suggest that ‘mint humbugs’ was the intruder. One possibility for this poor classification accuracy is that participants did not have enough context to interpret them correctly. In the *afternoon tea* topic, the top 5 items were predominantly fresh and ‘staple’ foods (e.g. Milk, Bananas, Danish sliced white bread). Thus, seeing a packet of sweets (i.e. ‘humbugs’) among this fresh food may have appeared unusual. An analogous issue arises with the *low maintenance cooking* topic. Each topic is a probability distribution over thousands of products, so perhaps it is not surprising that a small sample of products could be ambiguous.

Another possibility that is more core to our theory is that individual differences in experience may help explain some of these confusions. For example, the poor classification of the *afternoon tea* topic may have been driven by the fact that most British people no longer regularly engage in this ritualistic activity. If experience shapes people’s mental concepts, then we would expect representations of certain products to vary between demographics. Supporting this view, consumers from Northern Ireland had an average probability for the *Northern Ireland* topic 7.5 × higher than the average across all regions. The fact that the model was able to recover such strong regional differences in consumers suggests that it should be sensitive to other individual differences in people’s experience of the world.

## Classifying Individual Consumers by Their Experienced Topics

If the topic model proposed in this paper is representative of people’s semantic categories, then it should also be able to uncover individual differences in their representations. To test this assertion, we used logistic regression to predict (5-fold cross-validation) self-reported age^5^, region^6^ and gender using consumers’ mean LDA probabilities over baskets as the predictors.

### Method

#### Data

The feature set for the supervised models comprised of the training set topic probabilities output by LDA, averaged at the customer level. These features were then used to predict customers’ self-reported age (discretized into 18–29, 30–44, 45–59 and 60+), region (binarized into England vs. regional (i.e. Scotland, Wales and Northern Ireland)) and gender. These self-reported values had been gathered by the marketing panel described in the “Evaluating Topic Coherency with Typical Consumers” section during the last 3 years. The final modelling set contained data from 28,122 customers.

#### Model

To find the best performing model, we performed a grid-search between *λ* values of 0.1 to 1.0. Model selection was performed using the average predictive performance over 5 cross-validation folds. Baselines were calculated by predicting the majority class in each fold.

The age model was evaluated according to the average classification accuracy across the four classes. The gender and region models were binary classification problems, and thus evaluated in terms of the area under the ROC curve (AUC).

Results showed that the models were able to predict age with an accuracy of 48.51%, region with an accuracy of 58.34% and gender with an accuracy of 57.17%, considerably higher than the guessing baselines of 36.85%, 50.00% and 50.00%, respectively.

## General Discussion

Rather than being solely defined by intrinsic features (Plato 1973), concepts gain meaning through their interaction in the real world. Support for this notion comes from laboratory studies demonstrating that object interactions alter how people conceptualize objects (Jones and Love 2007) and from large-scale corpora analysis of text (e.g. newspaper articles) that extract meaning from word co-occurrence patterns. However, none of these previous investigations involve individuals engaging in unfiltered, goal-driven, real-world interactions with objects. Under such conditions, can meaningful conceptual organization be recovered from human activity patterns?

We tested this possibility by considering the shopping patterns of thousands of UK consumers. Using LDA, we found that the pattern of consumer purchases was highly revealing of people’s conceptual organization of these products. Topics ranged from specific and goal-driven (e.g. ingredients for a stir-fry) to very general (e.g. cooking from scratch). Interestingly, the topics tended to be goal-directed and situational, which is consistent with the notion that much of human conceptual knowledge is defined relationally and tailored to support action (Murphy and Ross 1999; Ross and Murphy 1999; Schank and Abelson 1977). The situational nature of certain topics was reflected in their increasing prevalence during certain times of the year, such as the *Christmas* topic in December and the *Summer salad* topic in the Summer.

The psychological reality of the 25 LDA topics we found was confirmed by two studies, one involving retail experts and one involving everyday consumers. The experts, who were blind to the purposes of this research, agreed with our labeling of the topics. The novices were able to identify an intruder product among an array of products from the same topic. These results indicate that the topics uncovered by human activity patterns are both comprehensible and coherent.

If concepts gain meaning through the actions we take, then individual differences in experiences should be reflected in differences in conceptual organization. In support of this conjecture, topic prevalence varied across geographic regions. In our study of everyday consumers, poor performance for the topic *afternoon tea* may reflect that today’s typical British consumer differs from past caricatures. Consistent with the idea that different types of people will have different topic experiences, we were able to predict basic demographic information about consumers from their topics mix (i.e. which topics best characterized their purchasing behaviour). One avenue for future research is to develop, apply, and evaluate topic models in which individuals organize into higher-level groups that can vary in terms of topic prevalence or even topic composition.

Taxonomic and thematic cross-classifications of food are typically measured in free-sorting tasks, where participants must sort food items into groups (Ross and Murphy 1999; Murphy 2001). Whilst originally suggesting that people have a bias towards sorting food taxonomically, more recent, larger-scale sorting tasks have suggested that people have a thematic bias (Lawson et al. 2017), suggesting that taxonomic bias is an artifact of a small initial set size. The large-scale analyses presented here give further credence to this claim, which is notable, given that supermarkets tend to arrange food taxonomically. Another likely cause of thematic bias is that grocery retail data reflects more goal-directed behaviour. For example, people may visit solely in order to purchase ‘food for now’, which emerged as a topic in our model. An outstanding question however is how people recruit these different representations over the course of a large shopping trip, as they complete several sub-goals. One possibility is that people recruit taxonomic and thematic representations hierarchically, using thematic representations to identify which ingredients to combine (e.g. for a salad) and taxonomic representations to identify the most suitable version of a given ingredient (e.g. best variety of tomato). Future research may wish to investigate this interaction in more detail.

We hope that combining large-scale analyses of grocery retail data with controlled experiments in this way will continue to reveal unique and important insights about the content and use of conceptual structures. Importantly, the work presented here has shown that people’s representations of food in the wild tend to be more thematic and goal-directed, but will vary depending on individual differences in experience. It is notable that such revealing representations can be learned on grocery retail data with relative ease. These results therefore support an emerging trend for using naturally occurring data sets to evaluate psychological claims (Goldstone and Lupyan 2016). Excitingly, this grocery data did not require heavy pre-processing, nor did it require any domain expertise to prepare. Future research can now use the foundation laid in this paper to investigate how semantic representations relate to other psychological phenemonena observed, as language corpus models have historically (Griffiths et al. 2007). For example, in future research, we intend to investigate how semantic representations influence people’s ability to effectively remember items in shopping lists, thereby investigating how semantic and episodic memory interact in the wild.

One interesting question is whether shopping activity changes conceptual organization or conceptual organization drives shopping behaviour. Our results cannot definitely answer this question, but the likely answer is that the influence is bidirectional, much like how choices follow from preferences and preferences to a degree follow from choices (Riefer et al. 2017). For example, having a concept like *stir-fry* should cause certain items to be purchased together to fulfill the common goal. Likewise, ingredients in the same dish may come to be viewed as more similar over time, consistent with laboratory studies that find that linking objects makes them more similar (Jones and Love 2007). One practical ramification is that recommender systems (Vasile et al. 2016; Christidis et al. 2010) using techniques related to our own may themselves shape conceptual organization.

What is clear is that conceptual organization is deeply tied to extrinsic relationships and that meaning can be seen as a byproduct of an element’s role within a larger system or web. Indeed, the insight behind Google’s PageRank algorithm is that web pages should be prioritized to the extent that they are central within a link graph (Page et al. 1998). Prior to PageRank, the exact same algorithm was developed in Psychology to explain why certain features of concepts are more central than others within a concept web (Love and Sloman 1995). Whether the system is human or artificial or the domain involves natural language or shopping behaviour, meaning can be inferred, and perhaps arises, from relations among elements embedded within a larger system.

## Electronic supplementary material

Below is the link to the electronic supplementary material.

(PDF 348 KB)

## Footnotes

1 More detail about the model fitting procedure can be found in the methods section

2 This same subset of 10 topics was considered in the empirical studies of retail experts (described in the “Evaluating Topic Labels with Retail Experts” section) and typical consumers (described in the “Evaluating Topic Coherency with Typical Consumers” section). Analyses was limited to a random subset of topics to reduce the overall survey time and thereby maximize the number of people that could take the respective surveys.

3 See Supplementary for more information about how relevance was calculated

4 More information about the ranking procedure used is available in the Supplemental

5 Discretized into 18–29, 30–44, 45–59 and 60+

6 Binarized into England vs. regional (i.e. Scotland, Wales and Northern Ireland)
