# Supplementary material for: Conceptual Organization is Revealed by Consumer Activity Patterns
Source: Comput Brain Behav. 2019 Oct 7;3(2):162–73. doi: 10.1007/s42113-019-00064-9 (PMC7235073; doi:10.1007/s42113-019-00064-9)
Supplement: Supplementary file 1 — (PDF 348 KB) [file 42113_2019_64_MOESM1_ESM.pdf]

Supplementary Information

0.1 Latent Dirichlet Allocation

For this project, we used a topic model known as Latent Dirichlet Allocation (LDA) (Blei et al., 2003). LDA is a generative probabilistic model that groups data into K unobserved topics. In the case of this project, baskets are represented as random mixtures over unobservable topics. Topics are then characterized as a mixture over N distinct products and D baskets. The generative process used by LDA can be described as follows:

1. For each topic $k \in \{1, ..., K\}$:
   - Choose a distribution over products $\phi_k \sim Dir(\beta)$.
2. For each basket $d \in \{1, ..., D\}$ in the collection c:
   - Generate a vector of topic probabilities: $\theta_d \sim Dir(\alpha)$
   - For each product $w_{d,n}$ in basket d:
     - Generate a topic assignment: $z_{d,n} \sim Multinomial(\theta_d)$
     - Draw a product $w_{d,n} \sim Multinomial(\phi_{z_{d,n}})$

Where β and α are hyperparameters that determine the concentration of the Dirichlet prior placed on the topics' distribution over products ϕ and the baskets' distribution over topics θ, respectively. The latent variables ϕ_k and θ_d can then be inferred using an iterative learning algorithm, such as expectation maximization (Blei et al., 2003).

0.1.1 Topic inspection and labelling

For each experiment, we calculated model *perplexity* on the training set and a held out test set. The perplexity of a model on that collection is defined as:

$$Perplexity(c) = exp\left\{\frac{-\sum_d log\, p(w_d)}{\sum_d N_d}\right\}$$

Where $p(w_d)$ is the probability of a product w in a basket d and N_d is the number of products in a basket. Perplexity can be useful to monitor during model training to ensure that the algorithm is converging on the training set and generalizing to unseen data. However, some have argued that perplexity and human interpretation are uncorrelated or even negatively correlated (Chang et al., 2009).

Given that interpretability is a fundamental goal of this research, we decided to test it more directly. In particular, we tested to see whether the topics were interpretable to humans and could identify known patterns in historic purchasing data. We now discuss this in more detail.

0.1.2 Calculating product relevancy for a topic

One conventional approach to interpreting topic models is to rank items (i.e. products) within a topic and manually inspect those with the highest probabilities. One can look for similarities between items with high probabilities to understand whether there is a key theme that binds them together.

A major issue with the traditional approach to interpreting topic models is that the probability of an item given a topic ϕ_{kw} can be biased positively in favour of

Table 1 The labels given to each of the 25 topics. Size is defined as the number of products that had the highest probability of belonging to the respective topic over the total number of products in the corpus. Asterisks indicate that they were surveyed in studies A and B.

Topic label	Size
Loose fruit and veg *	18.8%
Young children's shop	18.0%
Own brand shop	17.5%
Cooking from scratch *	16.9%
Snacks	16.4%
Cheapest option *	15.4%
Home baking	14.9%
Exotic cooking from scratch	14.5%
Afternoon tea *	14.5%
Quick to prepare meals	13.9%
Branded store cupboard	13.9%
Summer salad *	13.2%
Summer fruits	12.6%
Low maintenance cooking *	12.5%
Low calorie options *	11.3%
Party snacks	9.3%
Christmas *	7.3%
Northern Ireland	6.3%
Delisted Products	5.8%
Cat lover	1.3%
Stir fry *	1.1%
Own brand family party	1.0%
Food for now *	1.0%
Eating from tins	0.5%
Desserts	0.2%

more frequently occurring items within the corpus (Taddy, 2012). The validity of this traditional approach was therefore significantly limited, particularly given that we did not filter high-frequency products (i.e. stop items) from our dataset.

To overcome issues with biased item probabilities, we explored two additional measures for determining the pertinence of items within a topic; *lift* and *relevance*.

The lift measure (Taddy, 2012) is defined as:

Lift is therefore a ratio of an item w's probability within a topic k (i.e., $p(w_k)$) to its marginal probability across the corpus p_w. Whilst this helps to diminish the impact of overall token frequency, some have argued that the measure is noisy (Sievert and Shirley, 2014). In particular, it can give overly high rankings to items that occur very rarely within the corpus. Indeed — during our manual inspection of the topics — we found this to be the case, limiting our ability to interpret the topics' meanings.

To overcome this problem, Sievert and Shirley (2014) proposed the *relevance* metric, which is defined as:

$$r(w, k|\lambda) = \lambda \, log \, p(w_k) + (1 - \lambda) \, log \left(\frac{p(w_k)}{p_w} \right)$$

where λ is a free parameter that determines the weight given to the item w's probability within a topic k relative to its lift (measured on a log scale). If one sets $\lambda = 1$ then $r(w, k|\lambda) = log\, p(w_k)$. Alternatively, if $\lambda = 0$ then $r(w, k|\lambda) = log(lift(w, k))$. Thus, the benefit of this metric over lift is that it's possible to blend the probability of an item given a topic with lift. The metric's authors recommend using $\lambda = 0.6$ to maximize human interpretability, which is the value that we kept throughout all analyses (Sievert and Shirley, 2014).

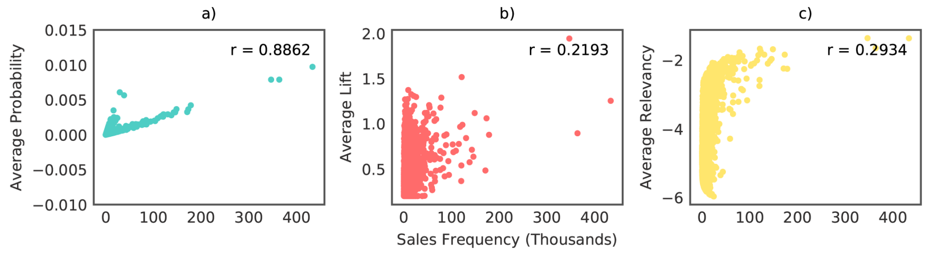

Fig. 1 The relationship between item frequency within the corpus and a) item-topic probability, b) lift and c) relevancy

The data displayed in Figure 1 plot the relationship between item corpus frequency and each respective metric. As discussed, results indicate a strong correlation between item probabilities and frequency ($r = 0.8862$). The lift metric ($r = 0.2193$) and relevance metric ($r = 0.2934$) considerably reduce this correlation.

After manually inspecting the topics with each of the three measures, we agreed that relevance provided the best measure of item salience within a topic. We therefore used this to help determine topic names.

0.1.3 Initial topic labelling

When labelling the topics, the authors inspected the relevancy scores of each item within each topic, considered the most relevant items. Table 1 shows the topic labels along with the relative size of each topic within the corpus.

Figure 2 depicts the most popular products within the corpus. As is with most retailers (and word usage in language), the data is highly right skewed. This suggests that there are a small number of products that are purchased across many baskets and a long tail of products that are less popular.

0.2 Label confusion by retail experts

In the retail expert study, errors in labeling appeared sensible. Namely, the most popular alternative labels tended to be related to the original topic (see Table 2). For example, the most popular alternative label to the *cooking from scratch* table was *loose fruit and veg*; both topic labels pertain to ingredients that need to be prepared before consumption.

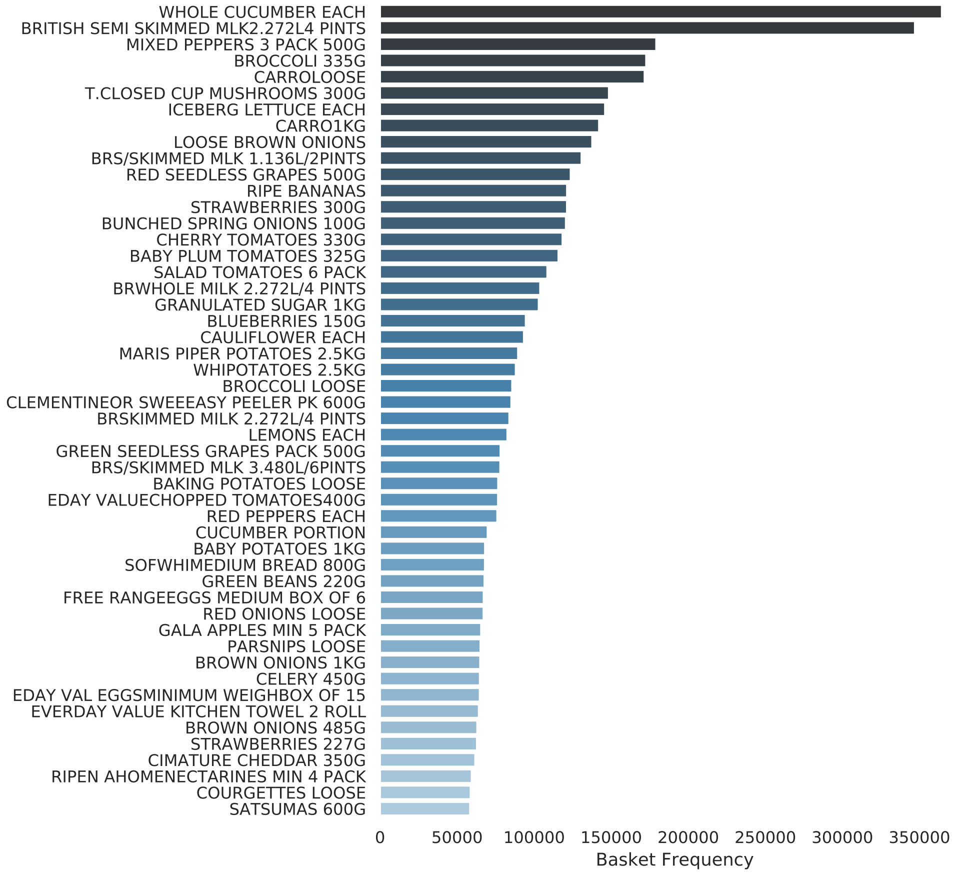

Fig. 2 The top 50 most frequently occurring items across baskets within the corpus. Note that brand names have been removed.

0.3 Topics by day of week

In addition to monthly trends, the proposed topic labels are also indicative of different weekly trends in purchasing habits. In particular, we hypothesized that topics indicative of longer preparation times (e.g. *loose fruit and veg*) or a special weekend occasion (e.g. *afternoon tea*) would be more likely to occur on or just before the weekend. Contrasting, we hypothesized that topics indicative of impulse purchasing (e.g. *food for now*) or stocking up for the long-term (e.g. *branded store cupboard*) would not vary much across the week.

References

Blei DM, Ng AY, Jordan MI (2003) Latent Dirichlet Allocation. Journal of Machine Learning Research 3:993–1022, DOI 10.1162/jmlr.2003.3.4-5.993, 1111.6189v1

Table 2 A summary of mislabelling errors made by retail experts during the topic labelling task (*** indicates a proportion significantly different ($p < .001$) from the random baseline of 25.00% (1 of 4))

Topic label	Proportion correct	Most confused topic	Most confused frequency
Cheapest option	0.98 ***		0
Food for now	0.961 ***	Low maintenance cooking	2
Stir fry	0.961 ***		0
Low calorie options	0.961 ***		0
Christmas	0.961 ***	Food for now	2
Afternoon tea	0.961 ***	Cheapest option	2
Loose fruit and veg	0.922 ***	Cooking from scratch	3
Summer salad	0.882 ***	Cooking from scratch	3
Cooking from scratch	0.784 ***	Loose fruit and veg	8
Low maintenance cooking	0.725 ***	Food for now	7

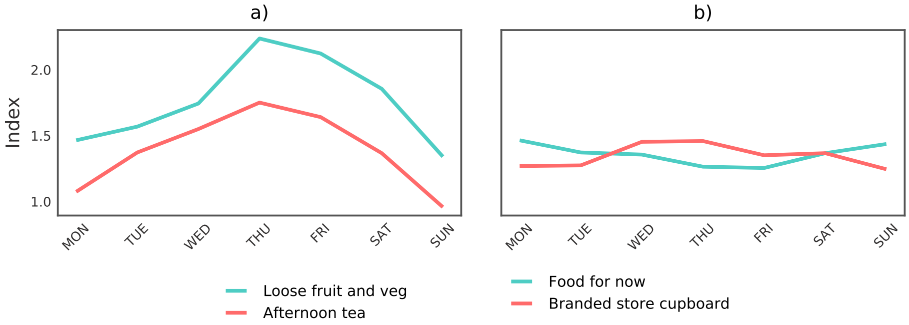

Fig. 3 The proportion of baskets with a given topic label on each day of the week, divided by the weekly mean average across all topics. Plot a) shows that food requiring longer preparation times (i.e. *loose fruit and veg*) or eaten specifically during weekend occasions (i.e. *afternoon tea*) are more likely to be bought on Thursday or Friday. Plot b) indicates that impulse purchases (i.e. *food for now*) or food that tends to be stored away (i.e. *branded store cupboard*) does not vary in popularity over the week.

Chang J, Gerrish S, Wang C, Blei DM (2009) Reading Tea Leaves: How Humans Interpret Topic Models. Advances in Neural Information Processing Systems 22 pp 288—-296, DOI 10.1.1.100.1089

Sievert C, Shirley K (2014) LDAvis: A method for visualizing and interpreting topics. Proceedings of the Workshop on Interactive Language Learning, Visualization, and Interfaces pp 63–70, DOI 10.1.1.100.1089

Taddy Ma (2012) On Estimation and Selection for Topic Models. In: Proceedings of the Fifteenth International Conference on Artificial Intelligence and Statistics, 2003, pp 1184–1193
